# The evaluation of a brief ICBT program with therapist support for individuals with gambling problems in the context of a gambling helpline: a randomized pilot trial

**DOI:** 10.1186/s40814-023-01257-7

**Published:** 2023-02-17

**Authors:** Håkan Wall, Kristoffer Magnusson, Clara Hellner, Gerhard Andersson, Nitya Jayaram-Lindström, Ingvar Rosendahl

**Affiliations:** 1 Department of Clinical Neuroscience, Centre for Psychiatry Research, Karolinska Institutet, Solna, Sweden; 2 Stockholm Health Care Services, Stockholm County Council, Norra Stationsgatan 69, 11364 Stockholm, Sweden; 3 Department of Behavioral Sciences and Learning, Department of Biomedical and Clinical Sciences, Linköping University, Linköping, Sweden

**Keywords:** Gambling helpline, Intervention, Pilot trial

## Abstract

**Background and aims:**

Gambling helplines are a natural way of first contact for individuals with gambling problems. However, few studies have evaluated the feasibility and effectiveness of brief interventions in a gambling helpline. To reduce this knowledge gap, this study evaluated the feasibility of an online cognitive behavioral therapy (ICBT) program in the context of a gambling helpline as a first step towards a full-scale RCT.

**Design:**

This is a two-group parallel randomized controlled pilot trial where the participants were randomized to either a brief four-module ICBT program (*n* = 22) or a control group (*n* = 21). Participants were followed up weekly during the intervention, post intervention, and 6 weeks upon completion of intervention.

**Participants:**

A total of 43 self-identified individuals with gambling problems (scoring 3 or more on the Problem Gambling Severity Index) were recruited via the Swedish national gambling helpline, 59% females, mean age 43.7 years.

**Measurements:**

Feasibility of the procedure and intervention (i.e., recruitment pace, attrition, program engagement, and satisfaction) were the primary outcomes; treatment effect (net gambling losses) was the secondary outcome.

**Results:**

Approximately 2 participants per week were randomized, and retention was low, with 47% lost to follow-up at the 6-week follow-up time-point. Most participants engaged in the online modules (86%) and rated their overall satisfaction with the program as high (7.5 out of 10). Both groups decreased their weekly gambling losses at both follow-up time-points, but the between-group comparisons were inconclusive.

**Conclusion:**

It is not advisable to conduct a full-scale RCT based on the results from this pilot study. Future studies in a gambling helpline should consider interventions that are more suited to be incorporated in a gambling helpline and identify ways to increase participant engagement.

**Trial registration:**

The study was retrospectively registered on ClinicalTrials.gov (ID: NCT04609007, 29/10/2020).

## Introduction

Gambling helplines have been a viable first way of contact for concerned gamblers in the last couple of decades. The scope of the help offered varies by country and jurisdiction. For example, some helplines offer crisis management and referrals to face-to-face treatment, whereas others offer brief interventions with different theoretical backgrounds to curb the gambling problems [1]. However, little focus has been paid on understanding the feasibility and effectiveness of offering brief interventions in the context of a gambling helpline, and most research so far has focused on describing those who make contact [2–7].

To date, only one study has evaluated the effectiveness of brief telephone interventions in the context of a gambling helpline. In a study conducted in New Zealand, helpline callers were randomized to one of four treatment arms: treatment as usual (TAU), a single session of motivational interviewing (MI), a single session of motivational interviewing plus a workbook or a single session of MI plus a workbook, and up to four MI-booster sessions over 6 months. The authors found that no treatment arm was superior compared to another. However, all participants showed large reductions in days gambling and money lost to gambling at the 12-month follow-up. Of interest, the retention rates were high throughout the study; 81% remained in the study at the 3-month follow-up and 64% at 12-month follow-up [8].

At the gambling helpline in Victoria, Australia, in a pilot study, the feasibility and efficacy of a brief SMS intervention were evaluated. A group of 198 gamblers were randomized to one of two treatment arms, 12 weeks of SMS with tips on how to change one’s gambling habits, or the helpline’s ordinary e-health services (treatment as usual, TAU). The SMS service consisted of 2 SMS per week, one SMS containing tips for behavioral change and one serving as a prompt to give feedback if the tip was helpful or not. At the 12-week evaluation, both groups spent less time and money on gambling and displayed reduced problem gambling severity. However, no between-group effects were found. Regarding feasibility outcomes, out of the 2482 gamblers who were invited to join the study, 249 (10%) were assessed for eligibility. In general, the retention was poor; at the 12-week follow-up, 39% of the participants remained in the study [9].

Although a couple of studies on brief interventions have been conducted in a helpline setting, most of them have been evaluated in non-helpline settings. Limited evidence has been found for self-assessment and workbooks: one study found that feedback on gambling habits without normative comparisons was superior to feedback with normative comparisons for time spent on gambling, but not for money spent on gambling [10]*.* In another study, receiving a workbook based on relapse prevention was found superior to a waitlist condition with regard to money spent on gambling [11], but it does not seem to matter if the workbook is portioned out over time or if all material is offered at one time-point [12]. Furthermore, at an online poker website, normative feedback was compared to a workbook condition, with or without guidance among non-help-seeking problem gamblers where 97% dropped out from the study, and all groups reported negative outcomes [13]. There is also some evidence for MI offered by phone. However, it remains unclear if offering additional workbooks or booster sessions improves the outcomes [14–17]. When it comes to treatment of individuals with gambling disorders, several meta-analyses have shown that cognitive behavioral therapy (CBT) is effective, at least in the short-term perspective [18–20]. Moreover, offering CBT via the Internet (ICBT) can be as effective as face-to-face treatment for several psychiatric and somatic disorders [21]. In a gambling context, ICBT has shown to be effective in mitigating the negative effects of problematic gambling. In Sweden, Carlbring and Smit showed that ICBT (eight online modules in combination with weekly telephone support) was superior to a waitlist control concerning PG severity, and that the positive outcomes were maintained for up to 3 years [22]. Similar ICBT programs have been evaluated in outpatient settings in Finland and Norway, with the results showing reductions in PG severity. However, given that no control condition was used in either study, no statements on the efficacy of the ICBT programs can be made [23–25]. A recent study compared guided to unguided ICBT. Between-group comparisons revealed that the guided ICBT was superior with regard to gambling frequency (gambling days per month). However, the authors found inconclusive results concerning problem gambling severity and gambling expenditures [26]. Entire self-help interventions based on CBT have also yielded some limited support [27–29]. The abovementioned studies show a heterogeneous pattern with regard to gambling-related outcomes, and it remains unclear if more extensive interventions are superior to briefer. Moreover, there is limited research on interventions offered in a helpline setting, and to our knowledge, no study has combined telephone counselling in a gambling helpline with a brief ICBT program with therapist support.

The Swedish gambling helpline is a national service which was formed in 1999. It was operated by a private company until 2010, and from 2011 and onwards, it has been operated by Stockholm Centre for Psychiatry Research, which is a part of Stockholm County Council and Karolinska Institutet. The helpline is funded by governmental means and has no affiliations with the gambling industry. The helpline offers anonymous counselling via telephone, email, and chat for individuals with gambling problems (IGPs) and their relatives and is opened on weekdays between 9 AM and 5 PM except for Mondays (9 AM to 9 PM) and Thursdays (11 AM to 9 PM). Via its webpage, it offers information (for both gamblers and relatives), online screening, and self-help without therapist support for IGPs. The telephone counselling is based on MI, and all counsellors are trained, supervised, and monitored to keep high MI standards. The helpline does not offer repeated calls; however, callers are prompted to call back to follow up if there is a need. On an annual basis, approximately 1000 gamblers contact the helpline for counselling, and an additional 1200 gamblers register an account at the online self-help program [30]. In general, those contacting the helpline experience quite severe gambling problems; at the helpline’s online screening, the average score on Problem Gambling Severity Index (PGSI) [31] was 15.5 points during the period 2015 to 2018 [32].

Since there is limited information on the feasibility of offering Internet interventions in conjunction to telephone counselling in a gambling helpline, a first step towards a full-scale RCT would be to conduct a pilot study primarily focusing on feasibility of the procedure and intervention. Consequently, the aim of the current pilot study was to evaluate the feasibility of a brief four-session ICBT intervention in conjunction to a helpline telephone counselling session. More specifically, the feasibility goals were to evaluate the following: recruitment, eligibility criteria, randomization, retention rate, adherence to the program, satisfaction with the program, and feasibility of the outcome measures. A secondary aim was to estimate the treatment effect.

## Method

### Study design

This study was a two-group parallel randomized controlled pilot trial where the participants were randomized to either a brief ICBT program with therapist support or a control group and where the users logged their gambling expenditures on a weekly basis. All participants were offered the helpline’s ordinary counselling prior to randomization. Participants were followed-up during the intervention and at two additional time-points, 6 and 12 weeks respectively, post randomization.

### Participants and procedure

**Fig. 1:**
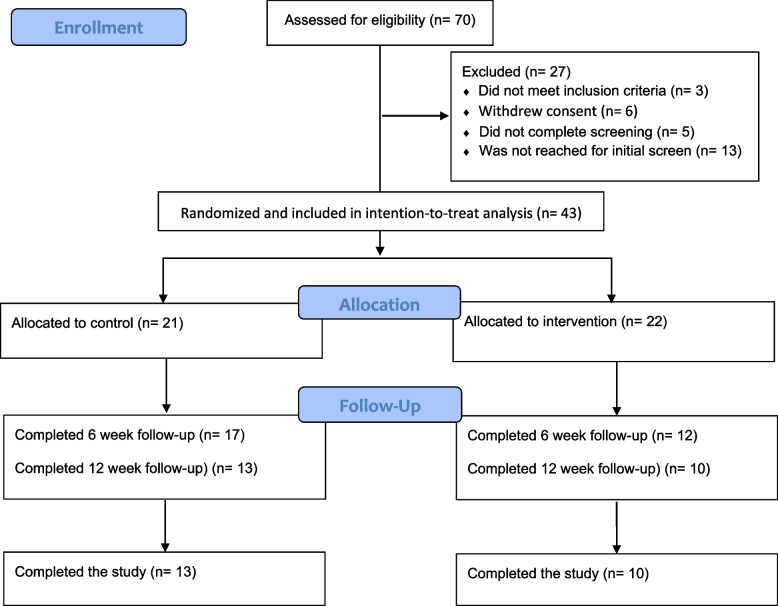
Participant flow

Participants were recruited via the gambling helpline telephone counselling and webpage between 2017-12-10 and 2018 April 20. Potential participants were redirected to a study-specific webpage. Although the goal was to recruit participants via the helpline, the study-specific webpage could also be found via various search engines, and consequently, potential participants could find the study-specific webpage directly. At the study-specific webpage, participants could read about the purpose of the study, eligibility criteria, and the research group conducting the study and register interest to participate. To register interest to participate, participants had to fill out a valid email address and tick a box that they had read and understood the informed consent present at the registration page. Once a registration of interest was submitted, a link to a screening survey was sent to the specified email address. All assessments were done via online surveys; three email reminders were sent for each assessment. Prior to randomization, all participants received the helpline’s regular counseling. See Fig. 1 for participant flow. The goal was to recruit 20 participants to each study arm; the rationale for recruiting 40 participants was this number would be sufficient to evaluate the feasibility outcomes. The study was conducted in accordance with the Helsinki Declaration and was approved by the Stockholm Regional Ethics Board (ref: 2017/1063-31) and registered retrospectively on ClinicalTrials.gov (ID: NCT04609007, 29 October 2020, https://clinicaltrials.gov/ct2/show/NCT04609007).

### Screening

The screening survey consisted of the PGSI, the Consumption Screen for Problematic Gambling (CSPG) [33], the Patient Health Questionnaire (PHQ-9) [34], gambling status, and contact information. Once a participant had expressed interest in participating in the study and had filled out the screening survey, a licensed psychologist called the participant for an eligibility check. Individuals were excluded from the study on the following parameters: those who scored high on PHQ-9 (≥ 20 points) were asked about current contacts with psychiatric care, and those who did not have a current contact were encouraged to contact their GP. Participants who were assessed as too depressed to participate in the study were encouraged to contact their GP and were offered the helpline’s ordinary telephone counselling. Participants who scored one point or more on PHQ-9 item 9 regarding death wish and self-harm were screened for suicidality. Initially, the threshold for suicidality was set to no suicidal ideation at all, but since almost all initial participants displayed some level of suicidal ideation during their lifetime, the exclusion criteria were revised via an amendment to the Swedish ethics review board, to only exclude those with active plans to commit suicide.

### Eligibility criteria

Eligibility criteria were set to resemble the typical helpline caller, i.e., individuals with severe gambling problems and experience of poor mental health (including suicidal ideation). In addition, the participants had to be as follows: 18 years or more; score 3 points or more on the PGSI; not suicidal (i.e., no suicide plans); not severely depressed, or psychotic; and not in need of emergency assistance regarding housing or money issues. Moreover, the participants had to be able to understand Swedish at a level that enabled them to access the written online material in the surveys and the online modules. As mentioned previously, those not meeting the eligibility criteria were excluded from the study and offered the helpline’s ordinary telephone counselling or were referred to healthcare services.

### Assessments

See Table 1 for information on assessment time-points and outcome measures used at each time-point.

**Table 1:** Outcome measures at each time-point

Outcome measure	Time-point				
	Screening	Baseline	Weekly	Posttreatment	6 weeks
CSPG	*				
PGSI	*				*
TLFB-G		*	*	*	*
NODS		*		*	*
GUS		*		*	*
GASS		*		*	*
PHQ-9	*			*	*
GAD-7		*		*	*
AUDIT-C		*		*	*
DUDIT-C		*		*	*
WHQQOL-bref		*		*	*

PGSI used a 3 months’ time frame. Weekly refers to weekly measures during treatment (for 6 weeks)

### Randomization

To get balanced groups, block randomization with randomly varying block sizes (1–4), i.e., 2 to 8 participants per block, was used [35]. The R-package blockrand [36] was used for this purpose. A person outside the research group was responsible for the randomization and allocating participants to the different study arms. Neither participants, therapists, nor the statisticians were blinded to treatment allocation.

### Therapists

One licensed psychologist (first author) and one helpline counsellor (certified MI trainer) with experience in CBT treatment acted as therapists. Regular meetings were held to ensure that both therapists provided feedback consistent with CBT and on the same level of detail.

### Statistical methods

Gambling expenditure data, which has an excess of zeroes, was analyzed using a two-part model. This model is flexible enough to account for the large number of reports of zero losses while also allowing for the fact that gambling losses are typically heavily right skewed [37]. This is achieved by combining two generalized linear mixed-effects models (GLMM)—a logistic GLMM for the zero part and a skewed continuous (G)LMM for the nonzero expenditure. Since we were interested in the overall reduction in expenditure, the model is re-parametrized so that change in expenditure refers to the overall expenditure including zeros [37, 38]. The R-package brms [39], which is a higher-level interface for the probabilistic programming language Stan [40], together with a custom brms family for a marginalized two-part lognormal distribution was used to fit the model [37]. For all other outcomes, ANCOVAS were used. All ANCOVA models were adjusted for the baseline value. No imputations were performed due to the small sample size and few follow-up time-points.

### Measures

To assess recruitment pace, the average number of individuals who reported interest to participate in the trial and the average number of randomized participants per week were calculated. To assess adherence to the intervention, descriptive statistics on accessed modules was calculated. To assess retention, the loss to follow-up at each time-point was calculated. Three items at the end of each online module was used to rate the helpfulness, satisfaction, and overall satisfaction with each module, and 10 items rating various aspects of satisfaction with the intervention were administered during the posttreatment follow-up; see Table 3 for more information on the various items.

For the baseline assessment, a survey including background information (e.g., gender, marital status, education, and occupation); game types engaged in the previous month; game type(s) associated with problematic gambling; mode of access (i.e., at a brick-and-mortar venue/shop, via smartphone, via tablet, or via laptop); gambling-related debt; gambling goal (quit, quit problematic game types, limit gambling); and importance and confidence to change gambling habits was constructed. Primary outcome for the quantitative part of the study was net losses due to gambling, expenditure data was collected via a timeline follow-back procedure (TLFB-G) where the participants were encouraged to fill out net gambling losses in an online calendar for each day during a specific timeframe [41]. Both study groups filled out the TLFB-G calendar (net losses) online once a week (rated net losses for each day the past 7 days) for six consecutive weeks. Secondary outcomes for the quantitative part of the study were as follows: the nine-item *Problem Gambling Severity Index* (PGSI), which measures gambling problems in the general population. The total score ranges from 0 to 27 points, and a score above 8 points indicate gambling problems [31]. The PGSI has excellent psychometric properties [42]; this study however used a Swedish version which has not been validated. Furthermore, the original timeframe is set to 12 months, and this study used a 3 months’ timeframe. The three-item *Consumption Screen for Problematic Gambling* (CSPG) [33] was used to get a composite measure of frequency of gambling. The total score ranges from 0 to 13, and a score of 4 and above indicates gambling problems. In a validation study, the internal consistency for the scale was high (alpha = 0.93), and at the 4-points cutoff, the sensitivity was 1 and specificity 0.93 [33]. The scale was translated into Swedish for this study and is not validated in a Swedish context. *The NORC DSM-IV Screen for Gambling Problems* (NODS) [43] was used to measure the level of gambling problems. The scale consists of 17 items which are answered yes or no, and the total score ranges from 0 to 10, where each point indicates a DSM-IV criterion for gambling disorder. The scale has shown acceptable psychometric properties [44]. *Gambling Abstinence Self-Efficacy Scale* (GASS) [45] was used to measure self-efficacy in gambling situations. The scale consists of 21 items which are answered on a 6-grade Likert scale (0–5), and the total score ranges from 0 to 105. The scale has been validated on abstinent Canadian problem gamblers. The scale showed high internal reliability, and higher composite score predicted fewer gambling days among gamblers not in treatment but not for those in treatment [45]. The scale was translated into Swedish for this study and has not been validated in a Swedish context. *Gambling Urges Scale* (GUS) [46] was used to measure gambling urges. The scale consists of 6 items on a 7-grade Likert scale (0–6), and it showed good psychometric properties in a sample of community-recruited individuals. The scale was translated into Swedish for this study and has not been validated in a Swedish setting. *Alcohol Use Disorder Identification Test-Consumption* (AUDIT-C) was used to measure alcohol consumption. AUDIT was initially developed by the World Health Organization (WHO). AUDIT-C contains the 3 initial questions regarding consumption, and in a Swedish context, the scale performed excellent at detecting alcohol dependence and alcohol use disorder but not as good in detecting risk drinking. The score ranges from 0 to 12 points, and a score of 6 and above indicate alcohol use disorder among men and a score of 5 and above among women [47]. *Drug Use Disorder Identification Test-Consumption* (DUDIT-C) was used to measure illicit and prescribed drug consumption. DUDIT-C contains the first 3 questions regarding consumption of illicit or prescribed drugs from the DUDIT questionnaire. The scale has high internal consistency (alpha = 0.80) and is congruent with both ICD-10 and DSM-IV regarding hazard use/dependence of illicit substances [48].

Patient Health Questionnaire-9 (PHQ-9) was used to measure symptoms of depression and consists of 9 items on a 4-grade Likert scale, ranging from 0 “not at all” to 3 “almost daily.” The total score ranges from 0 to 27; 0–4 indicate minimal, 5–9 mild, 10–14 moderate, 15–19 moderately severe, and 20-–7 severe depression [34]. Generalized Anxiety Disorder 7-item scale (GAD-7) was used to measure level of anxiety. The total score ranges from 0 to 21, and 0–5 indicate minimal, 6–10 mild, 11–15 moderate, and 16–21 severe anxiety [49]. WHOQOL-BREF is an instrument developed by the WHO and measures quality of life. It contains 26 items and is a condensed version of WHOQOL-100. The scale has good psychometric properties and is valid in various cultural settings [50].

### Intervention

The ICBT program, which was a condensed version of the eight-module ICBT program by Nilsson et al. [51], consisted of 4 modules, which were offered on a weekly basis for up to a total time-period of 6 weeks; see Table 2 for description of module content.

**Table 2:** Description of module content

Module	Content	Length
1. Psychoeducation and goal setting	• Introduction to the program • Rationale for gambling problems • Goal setting and values compass	Pages: 6 Exercises: 3 Videos: 1 (1:31 min)
2. Understand your gambling	• Define gambling situations • Explore potential reinforcers • Plan alternative responses to gambling situations	Pages: 3 Exercises: 3 Videos: -
3. Gambling thoughts and urges	• Identify gambling thoughts and how to deal with them • Identify gambling urges and how to deal with them	Pages: 4 Exercises: 5 Videos: 1 (1:32 min)
4. Deal with relapses	• Lapse vs. relapse • Prevent future relapses	Pages: 4 Exercises: 5 Videos: 1 (1:20 min)

To facilitate adherence to the program, feedback to the participants was as contingent as possible on participant engagement in a module. Feedback to the clients was written in an encouraging MI style. At first log in to the ICBT-program platform, the participants had a welcome message where the therapist introduced him-/herself plus a brief description on how to get started with the program. Messages between client and therapist were sent via a secure message system within the online program platform. E-mails were only sent to prompt clients and therapists that they had new messages in the program platform message system.

## Results

Seventy individuals were assessed for eligibility, and a final sample of 43 were randomized to one of the two study arms; see Fig. 1 for participant flow. Females constituted 49% of the participants, and the sample mean age was 43.7 (*SD* = 11.9) years. Online slots were the most reported “most problematic” type of gambling (79%), the median duration of gambling being identified as a problem was 24.5 months (about 2 years), and the median debt due to gambling was 215,000 SEK (1 SEK ~ 0.11 USD). The most common treatment goal was “quit gambling” (*n* = 38, 90.5%) followed by “quit gambling on problematic game types” (*n* = 4, 9.5%). No one stated “limited/controlled gambling” as a treatment goal. Readiness to change was high, 9.77 (*SD* = 0.68) on a VAS scale from 0 to 10, and confidence in succeeding was intermediate, 6.95 (*SD* = 2.24) on a VAS scale from 0 to 10. See Table 3 for background and gambling-related characteristics.

**Table 3:** Demographic, gambling, and health-related variables at baseline

Variable	I-CBT (*N* = 22)	Control (*N* = 21)	Total (*N* = 43)
Age, mean (SD)	42.5 (13.90)	45.1 (9.54)	43.7 (11.90)
Female, *n* (%)	8 (36.4)	13 (61.9)	21 (48.8)
*Highest education level, n (%)*			
Elementary school	3 (13.64)	3 (14.28)	6 (13.95)
Secondary school	9 (40.91)	8 (38.1)	17 (39.53)
University	10 (45.45)	10 (47.62)	20 (46.51)
*Types of gambling, n (%)*			
Online slots	16 (72.7)	18 (85.7)	34 (79.1)
Online sports betting	3 (13.6)	2 (9.5)	5 (11.6)
Land-based sports betting	5 (22.7)	2 (9.5)	7 (16.3)
Horse betting	6 (27.3)	2 (9.5)	8 (18.6)
Lotteries/number games	3 (13.6)	5 (23.8)	8 (18.6)
Land-based EGMs	2 (9.1)	2 (9.5)	4 (9.3)
Land-based casino	2 (9.1)	0 (0)	2 (4.7)
Bingo (in venue or online)	1 (4.6)	2 (9.5)	3 (7.0)
Online poker	4 (18.3)	0 (0)	4 (9.3)
*Most problematic types of gambling, n (%)*			
Online slots	16 (72.7)	18 (85.7)	34 (79.1)
Online sports betting	2 (9.1)	1 (4.8)	3 (7.0)
Land-based sports betting	3 (13.6)	0 (0)	3 (7.0)
Horse betting	3 (13.6)	0 (0)	3 (7.0)
Lotteries/number games	0 (0)	0 (0)	0 (0)
Land-based EGMs	1 (4.6)	1 (4.8)	2 (4.7)
Land-based casino	1 (4.6)	0 (0)	1 (2.3)
Bingo (in venue or online)	0 (0)	1 (4.8)	1 (2.3)
Online poker	2 (9.1)	0 (0)	2 (4.7)
Previous participation in treatment, *n* (%)	3 (13.6)	4 (19.1)	7 (16.3)
Debt due to gambling, median	200,000 SEK	300,000 SEK	215,000 SEK
Months of problem gambling, median (IQR)	30.5 (73.8)	24 (31.5)	24.5 (53.5)
*Gambling and health-related outcomes, mean (SD)*			
TLFB-G lost/day (in SEK)	1095 (1728)	1221 (2442)	1155 (2073)
PGSI	20.5 (3.84)	21.7 (3.84)	21.1 (3.84)
NODS	6.2 (2.11)	6.2 (1.91)	6.2 (1.99)
CSPG	9.3 (2.35)	10.5 (1.86)	9.9 (2.20)
GUS	9.2 (8.10)	13.0 (9.12)	11.0 (8.70)
GASS	55.6 (15.70)	42.4 (27.3)	49.3 (22.70)
PHQ-9	16.5 (6.12)	14.8 (5.71)	15.7 (5.92)
GAD-7	10.5 (5.38)	9.5 (5.24)	10.0 (5.28)
AUDIT-C	3.7 (2.63)	3.4 (1.63)	3.5 (2.19)
DUDIT-C	0.09 (0.43)	0	0.05 (0.31)

### Recruitment

Recruitment to the study lasted for 141 days (approximately 20 weeks), which means that on average, 3.5 individuals per week registered interest to participate, and 2.15 participants were randomized per week. Most of the participants reported that they found information about the study via the gambling helpline’s webpage (*n* = 23, 53%) and the rest via a search engine (*n* = 11, 26%), the helpline’s telephone counselling (*n* = 6, 14%), or other sources (*n* = 3, 7%). Those who reported that they found information about the study via a search engine, or “other sources,” did not differ with regard to the gambling-related variables, gender distribution, or levels of anxiety or depression compared to those who found information about the study via the helpline’s telephone counselling or webpage. Out of 70 individuals who were interested to participate, a final sample of 43 were randomized, which gives a recruitment rate of 59%.

### Adherence to the program

The median number of opened modules was 4 (*IQR* = 3, *n* = 22, *M* = 2.7, *SD* = 1.61), 12 individuals opened all four modules, and three did not open any module at all. Seven out of 22 (31.2%) did not complete an assigned module, and the median number of logins to the platform was 7.5 (*IQR* = 7.50); two participants did not log in at all.

### Retention

Twelve in the intervention group and 15 in the control group completed all 6 weekly TLFB measures during the intervention. On average, the intervention group filled out 4.3 (*SD* = 1.80) weekly TLFB measures and the control group 5.0 (*SD* = 1.91). Some in the intervention group finalized the program in 5 weeks and, consequently, did not receive the 6th weekly follow-up. At the posttreatment follow-up, 12 from the intervention group and 17 from the control group remained in the study, and at the 12-week follow-up, 10 from the intervention group and 13 from the control group remained in the study, which gives a retention rate of 67% (29/43) at the posttreatment follow-up and 53% (23/43) at the 12-week follow-up.

### ICBT-program satisfaction

At the end of each module, the participants were asked to rate the module content. Module 4, relapse prevention, received the highest overall grade, 4.30 out of 5. Module 2, analysis of gambling situations, received the lowest grade, 3.79 out of 5. In the posttreatment questionnaire, the participants were asked to rate the ICBT program, and they were in general satisfied with the program and rated their overall satisfaction with the program to 7.5 out of 10. However, they rated the program’s helpfulness lower, 6.5 out of 10; see Table 4 for participant module and program ratings.

**Table 4:** Mean ratings of the separate modules (1–5) and overall ratings of the ICBT program

Module (*n*)	Helpful	Suitable	Overall rating
Module 1 (16)	4.1	4.3	4.2
Module 2 (13)	3.7	4.3	3.9
Module 3 (12)	3.9	4.3	4.5
Module 4 (9)	4.4	4.4	4.6
Overall ratings of CBT program (VAS scales 1–10, 1 lowest grade and 10 highest)			
How satisfied are you with the CBT program?		8.6	
How helpful do you think the program was?		8.1	
How comprehensive do you think the program was?		7.4	
How labor intensive do you think the program was?		6.0	
How helpful, overall, was the contact with the therapist?		8.0	
Was it easy or difficult to understand what the therapist wrote? (1 = very difficult, 10 = very easy)		9.0	
How easy was it to access and get started with the program?		8.8	
How did you experience the length of the program? (5 means just enough)		3.4	
Has the program been helpful to you in changing your gaming habits?		7.8	
Would you recommend the program to others who want to change their gaming habits?		9.1	

### Treatment goal

At posttreatment, 40% in the treatment group and 61.5% in the control group reported that they had reached their setup treatment goal “largely” or “completely.” The difference between the groups were non-significant (*p* = 0.41, Fisher’s exact test).

### Treatment effect

**Fig. 2:**
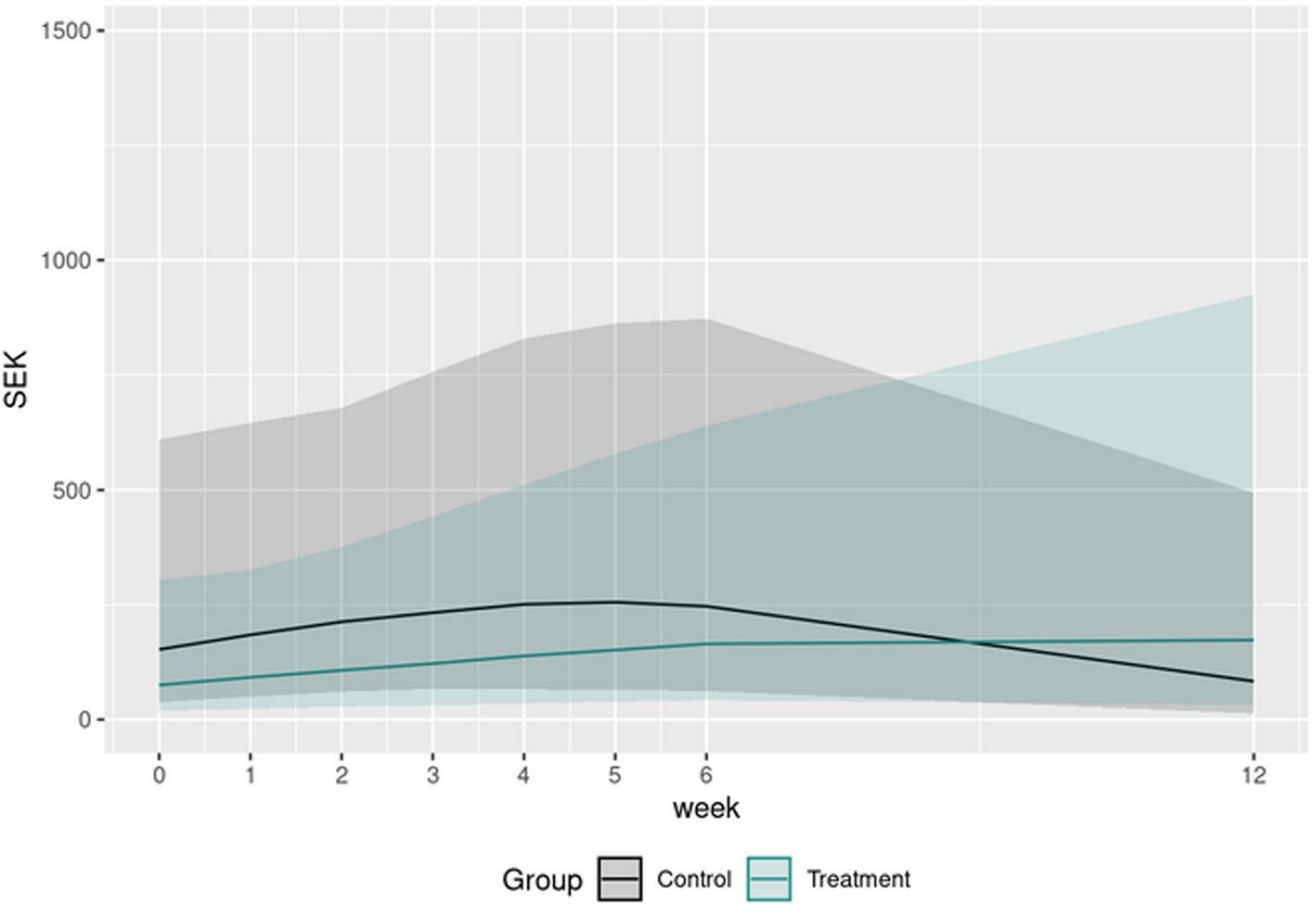
Average daily gambling losses in SEK per group from week 0 (first week of intervention) to week 12

Both groups reduced their gambling losses and global PG rating (NODS and PGSI), levels of anxiety and depression from baseline to the 6-week follow-up. However, all comparisons between the groups provided inconclusive results. For gambling losses, there was an indication that the intervention group increased their gambling losses during the follow-up period, and that they lost more money to gambling per day compared to the control group at the 6-week-follow up (79 SEK, 95% *CI* = −335, 790 SEK). See Fig. 2 for development of gambling losses over time and Table 5 for information on all quantitative outcomes. The strongest relationship between the gambling-related variables at the 6-week follow-up was found between NODS and GASS (*r* = −0.75, *p* ≤ .001), indicating that high levels of PG severity were strongly associated with low levels of perceived self-efficacy in mastering gambling situations. The correlation between PGSI and NODS was moderate (*r* = 0.67, *p* ≤ .001), indicating that the two outcome measures capture the same latent construct. Table 6 displays all correlations between the gambling-related outcomes at the 6-week follow-up.

**Table 5:** Treatment effects at posttreatment and 6-week follow-up. Negative coefficients indicate that treatment is superior to control

Estimated effects for ICBT and control group							
	Mean ICBT^a^ (SD)	Mean control^a^ (SD)	Diff.^a^	Coeff.	ES^b^	95% CI for ES	*p*-value
TLFB-G (SEK)							
Posttreatment	544 (1732)	356 (707)	188		0.67c	(0.11, 4.25)	
6 weeks	49.2 (72.9)	70.4 (95.1)	−21.2		2.01c	(0.71, 20.11)	
NODS							
Posttreatment	3.33 (3.75)	3.82 (3.41)	−0.49	0.37	0.18	(−1.09, 1.46)	0.77
6 weeks	2.44 (3.13)	2.00 (3.00)	0.44	−0.33	−0.16	(−1.85, 1.52)	0.84
PGSI							
6 weeks	10.0 (7.35)	9.2 (7.49)	0.8	1.01	0.26	(−1.25, 1.78)	0.72
GUS							
Posttreatment	6.6 (8.54)	6.7 (8.56)	−0.07	−0.7	−0.08	(−0.80, 0.64)	0.82
6 weeks	10.7 (12.30)	3.67 (11.00)	7.03	−7.79	−0.89	(−2.34, 0.55)	0.21
GASS							
Posttreatment	67.2 (28.00)	61.1 (33.50)	6.10	−1.38	−0.06	(−1.11, 0.98)	0.91
6 weeks	77.7 (20.30)	70.1 (37.10)	7.6	1.12	0.05	(−0.86, 0.96)	0.91
PHQ-9							
Posttreatment	8.6 (6.73)	9.2 (6.41)	−0.66	1.11	0.17	(−0.59, 0.93)	0.66
6 weeks	5.2 (6.18)	5.6 (5.53)	−0.34	2.27	0.37	(−0.51, 1.26)	0.38
GAD-7							
Posttreatment	6.8 (4.43)	8.5 (5.89)	−1.70	2.28	0.43	(−0.24, 1.09)	0.20
6 weeks	3.7 (4.23)	3.7 (5.89)	0	0.21	0.04	(−0.83, 0.91)	0.93
AUDIT-C							
Posttreatment	2.8 (2.37)	3.7 (2.82)	−0.90	0.41	0.18	(−0.48, 0.85)	0.58
6 weeks	2.1 (2.09)	2.2 (1.30)	−0.11	0.04	0.02	(−0.53, 0.57)	0.94

^a^Observed values

^b^Cohen’s *d*

^c^Multiplicative effect, < 1 treatment superior, > 1 control superior

**Table 6:** Correlation matrix for gambling-related variables at the 6-week follow-up, *N* = 18

Variable	GUS	GASS	PGSI	NODS
GUS	1	−0.43	0.62**	0.50*
GASS	−0.43	1	−0.63***	−0.75***
PGSI	0.62**	−0.63***	1	0.67***
NODS	0.50*	−0.75***	0.67***	1

*p ≤ .05, **p ≤ .01, ***p ≤ .001

## Discussion

### Recruitment

In this study, we found that it was possible to recruit participants to a brief online self-help program via a national gambling helpline’s webpage. However, to conduct a full-scale RCT, involving at least 100 individuals in each treatment arm would take approximately 1.8 years with the current recruitment pace (2.15 participants per week), and expecting a 50% dropout rate would mean that in total, 400 participants would be needed and additional 1.8 years of recruitment. In the only comparable study by Abbott et al. [8], they recruited approx. 6 participants per week for 1.4 years. In their study, they randomized the participants directly over telephone and offered the intervention immediately, which may have increased the willingness to participate in the study. Our study also had a lower recruitment pace compared to Rodda et al. [9], who recruited 4.8 participants per week for 12 months. To achieve a recruitment pace of approximately five participants per week, participants need to be recruited from more sources, and inclusion and randomization should be done when the participants make the initial contact to the helpline, regardless of channel (telephone, chat, or email). This will require that the organization be well prepared to manage the study for several years, and that the staff is trained to make the necessary assessments when the clients make contact.

### Eligibility criteria

The inclusion criteria were initially set to exclude all participants who reported any level of suicidal ideation. This was found to be too exclusive since most participants showed suicidal ideation when they filled out the screening surveys. However, during the follow-up interviews via telephone, several participants affirmed to have life fatigue, but none reported concrete plans to end one’s life. The exclusion criteria were changed to concrete suicide plans instead. This fitted the gambling helpline target population better, and the changed criteria is also in line with studies that have shown high rates of suicidal ideation among gamblers contacting a gambling helpline [5, 52, 53]. However, to offer clients with comorbid conditions, participation in this study may have affected attrition and how well the intervention was received.

### Retention and adherence to the program

Few individuals completed all the follow-up measurements; 29% and 44% were lost to follow-up at the posttreatment and 6-week follow-up, respectively. There was a trend that those in the intervention group dropped out to a greater extent compared to the control group, especially during the weekly TLFB measurements. This can be due to several reasons: firstly, there were more requirements in the intervention group; they both had to read and fill out exercises and fill out comprehensive weekly assessments of gambling losses compared to the control group that only had to fill out weekly gambling losses once a week. Secondly, the control group may have experienced the weekly measurements as an intervention per se, whereas the intervention group may have viewed it as an extra workload. We found in a previous study by our research group that logging gambling habits is not popular among gamblers [29]; this may also contribute to the high attrition during the weekly TLFB. However, similar attrition rates were observed in Rodda et al. [9], where more than 50% were lost to follow-up at the 4-week follow-up and 60% at the 12-week follow-up and in Jonas et al. [54] where more than 50% of the participants in a web-based intervention for gamblers were lost to follow-up after 12 weeks. Another way to get information on amounts spent on gambling during treatment could be to prompt the participants to reply on a SMS once a week where they estimate how much they have lost to gambling the previous week instead of asking participants to fill out a comprehensive TLFB calendar each week. In this study, we used automated emailing with three reminders to prompt the participants to fill out the follow-up surveys, this may not be optimal, and this could be combined with other methods, such as brief telephone calls, where non-responding participants are asked about the primary outcome. Regarding adherence to treatment protocol, most participants logged in to the system and engaged in the modules. The greatest drop was between the first and second module, where three participants dropped out and between the third and fourth module where two participants dropped out. None dropped out between the second and third module. Unfortunately, data on the amount of time spent in each module was not saved; however, the amount of text written in the different exercises varied between the participants; some wrote extensive answers to the different exercises, whereas others just wrote a few words. It is uncertain if this reflects time spent in the program or not, which is an apparent weakness in this study.

We have some suggestion on how retention in future helpline studies could be improved. In this study, the barrier to engage in the study was low; to raise the threshold by adding a pre motivation tool and only include those motivated to pursue with the treatment could have improved the retention rate. In the treatment program, we only communicated with the participants via direct messages. To add weekly telephone checkups could have improved the participant’s commitment and retention in the program. Furthermore, to increase follow-up rates, nudging the participants to answer the questionnaires could be one alternative, which should be combined with some sort of token economy where the participants are reimbursed incrementally for answering follow-up questionnaires. To maintain a long-term relationship with the participants, annual gifts and Christmas cards could be considered.

### Satisfaction with the program

In general, most participants were satisfied with the ICBT program as measured by a VAS scale from 0 to 10. That said, the analysis of gambling situations, which was considered the most important module by the research group since the ICBT program was built around analysis of gambling situations, received the lowest rating. This was unexpected and highlights the need to develop treatment programs like this in close cooperation with its end users. Future studies should engage end users in the program production phase, and a feasibility study focusing solely on program content and its suitability is recommended.

### Outcome measures

The follow-up surveys contained 140 and 138 items, respectively. Several questionnaires measured various aspects of the same construct. For instance, at the 6-week follow-up, five questionnaires measured various aspects of gambling (TLFB-G, NODS, GUS, GASS, PGSI). This may have been exhausting for the participants; fewer questionnaires on the same topic may be preferred. To exemplify, the correlation between NODS and PGSI was moderate (*r* = 0.67, *p* ≤ .001) at the 6-week follow-up; to omit PGSI would have reduced the burden for the participants without missing information on PG severity level. Moreover, initially, PGSI was intended to be used only in the initial screening but was added to the 6-week follow-up mainly for the reason that 3 months had passed, and it was possible to measure PGSI again. This decision provided an extra workload for the participants and should in retrospect have been avoided. Furthermore, we found that among those who did not fill out complete measurements at the 6-week follow-up, the common pattern was to quit when reaching the TLFB items. This indicates that it is difficult (or time consuming/boring) to fill out this type of calendar. A better way may be to ask about gambling losses in a single item, even though it may not give as high resolution as asking for daily gambling losses. On the other hand, most researchers aggregate the daily losses, which may speak in favor of letting the participants estimate their weekly losses as a composite score. Moreover, since it is well-known that gambling losses are difficult to estimate by the IGPs [55] and that decreased gambling losses may just reflect the fact that the IGPs has no money left to spend on gambling, it is not feasible to use it as the primary outcome in gambling treatment studies. It could also be argued that filling out a weekly TLFB calendar per se leads to an increased awareness of money lost to gambling, and that this awareness is a mediator between the treatment and the outcome. However, changed gambling behavior is important to measure in treatment studies like this one. An alternative could be measuring weekly problematic gambling episodes; this measure would of course also be open to interpretation by the IGPs but easier to measure compared to money lost to gambling, which could be used as a secondary outcome instead. As primary outcome in gambling treatment studies, we propose a measure based on the DSM-V criteria, although those questions are also open to interpretation by the study participants.

### Treatment effect

Both groups improved in all outcomes compared to the baseline measure. However, there was no support for any favorable outcomes for the treatment group in that all comparisons between the two study groups provided inconclusive results. A tendency towards increased gambling net losses during the follow-up period was observed for the treatment group. This tendency for the intervention group, although on a low level, could be connected to either the lower proportion participants in this group reaching their treatment goal or to the inherent problems reporting gambling losses mentioned above. However, given the low number of participants completing all follow-up measurements, this is mere speculation on our part. The result from this study is in line with other gambling intervention studies that have compared CBT to an active control group, Carlbring et al. [56] compared group-delivered CBT to face-to-face delivered MI, Nilsson et al. [51] compared ICBT to online behavioral couples’ therapy, and Casey et al. [57] compared ICBT to a monitoring and feedback intervention; none of these studies found the CBT intervention to be superior to the active control intervention regarding gambling outcomes.

The reasons for the inconclusive results in this study may be several; to begin with, the CBT program and the therapist feedback may not have been congruent with CBT or MI and therefore not effective. Another reason may be that those individuals with either too severe gambling problems and/or too severe comorbid conditions were included. This may have affected their possibility to assimilate the program content and implement the needed changes in their daily lives. Given that this study was a pilot study, no power calculation on how many participants were needed to find meaningful differences between the groups was done; nevertheless, the results do not indicate favorable outcomes for the ICBT group.

Gambling helplines have an obvious place in a society and play a significant role in bridging the treatment gap and as a promoter of further help-seeking in regular care. However, several questions need to be addressed; for instance, what kind of support should a helpline offer, and what is a desirable outcome in conjunction to a helpline contact? Today, MI is the preferred type of counselling method in many helplines, and the purpose of MI is to evoke the clients’ intrinsic motivation to change a dysfunctional behavior [58]. Given that, a positive outcome from a MI counselling session in a helpline should increased motivation and confidence in changing one’s problematic behavior. Actual change in the problematic behavior should be a secondary outcome. Furthermore, which types of beneficial long-term effects can you expect from a brief intervention, such as telephone counselling or a very brief online intervention in a gambling helpline setting, and how can you evaluate these effects given the high attrition from gambling treatment studies? Furthermore, the rapid technical development (apps, social media, etc.) affects what the clients expect from a help service, and self-developed online interventions can become obsolete in a brief time.

In general, we mean that gambling helplines today are underutilized. As an example, in Sweden, only a fraction of those who probably need advice and support call the helpline. One way to increase accessibility could be to offer more automated services such as a chatbot that can answer general questions such as referrals to treatment centers or how to self-exclude from gambling. We believe that gambling helplines should be built on a stepped care approach with services ranging from evidence-based information available online via self-help modules to extended telephone counselling, all depending on the level of help needed. These different services should be evaluated on a daily basis; even though helpline callers may not be randomized to different interventions, the information on how their gambling behaviors develop over time is valuable. For online interventions, it is possible to randomize individuals to different setups of an intervention (so-called A/B testing) to see which setup performs better. This way we can better understand how interventions in a helpline setting work for their end users both in the short- and long-term perspective.

### Strengths and limitations

The broad inclusion criteria are a strength in this study; the participants represent the helpline callers for which the ICBT program was developed. This study has some limitations that must be addressed: firstly, the extensive attrition during follow-up, which limits the validity of the findings. Secondly, we used several outcome measures that have not been validated in a Swedish context, which may compromise the results generalizability to other countries. Thirdly, due to the lack of measures based on the DSM-V criteria for gambling disorder in Swedish, we used NODS, which is based on the DSM-IV criteria. Although this is a limitation to this study, NODS has been used in several previous treatment studies in Sweden. Fourthly, we did not involve the end users (IGPs) in the development of the different online modules and the program setup; this should have been done as a first step preceding this pilot study and is also a limitation.

## Conclusion

It is possible to recruit individuals to an intervention study in a helpline setting. Nonetheless, given the recruitment pace in this study, it cannot be considered efficient to recruit participants via an external website. Recruitment should be in conjunction with the initial contact with the helpline. Participants were in general satisfied with the ICBT program, but attrition was high, and there was no indication of the intervention being superior to the control intervention. We suggest that future intervention studies in a helpline setting should focus on very brief interventions that require a minimum effort from the participants, and that outcomes, such as being better prepared for change or treatment seeking post the helpline contact, should also be measured to better capture the whole spectrum of relevant outcomes from a helpline contact.

## Data Availability

The datasets used and/or analyzed during the current study are available from the corresponding author on reasonable request.

## References

[1] Clifford G, Zangeneh M, Blaszczynski A, Turner NE (2008). The evolution of problem gambling helplines. In the pursuit of winning.

[2] Barry DT, Steinberg MA, Wu R, Potenza MN (2009). Differences in characteristics of Asian American and white problem gamblers calling a gambling helpline. CNS Spectr.

[3] Barry DT, Steinberg MA, Wu R, Potenza MN (2008). Characteristics of Black and white callers to a gambling helpline. Psychiatr Serv.

[4] Kim HS, Hodgins DC, Bellringer M, Abbott M (2016). Gender differences among helpline callers: prospective study of gambling and psychosocial outcomes. J Gambl Stud.

[5] Ledgerwood DM, Steinberg MA, Wu R, Potenza MN (2005). Self-reported gambling-related suicidality among gambling helpline callers. Psychol Addict Behav.

[6] Potenza MN, Steinberg MA, McLaughlin SD, Wu R, Rounsaville BJ, Krishnan-Sarin S (2004). Characteristics of tobacco-smoking problem gamblers calling a gambling helpline. Am J Addict.

[7] Potenza MN, Steinberg MA, McLaughlin SD, Wu R, Rounsaville BJ, O’Malley SS (2000). Illegal behaviors in problem gambling: analysis of data from a gambling helpline. J Am Acad Psychiatry Law.

[8] Abbott M, Hodgins DC, Bellringer M, Vandal AC, Palmer Du Preez K, Landon J (2018). Brief telephone interventions for problem gambling: a randomized controlled trial. Addiction.

[9] Rodda SN, Dowling NA, Knaebe B, Lubman DI (2018). Does SMS improve gambling outcomes over and above access to other e-mental health supports? A feasibility study. Int Gambl Stud.

[10] Cunningham JA, Hodgins DC, Toneatto T, Murphy M (2012). A randomized controlled trial of a personalized feedback intervention for problem gamblers. PLoS One.

[11] Oei TPS, Raylu N, Lai WW (2018). Effectiveness of a self help cognitive behavioural treatment program for problem gamblers: a randomised controlled trial. J Gambl Stud.

[12] Hodgins DC, Currie SR, El-Guebaly N, Diskin KM (2007). Does providing extended relapse prevention bibliotherapy to problem gamblers improve outcome?. J Gambl Stud.

[13] Luquiens A, Tanguy ML, Lagadec M, Benyamina A, Aubin HJ, Reynaud M (2016). The efficacy of three modalities of Internet-based psychotherapy for non-treatment-seeking online problem gamblers: a randomized controlled trial. J Med Internet Res.

[14] Boudreault C, Giroux I, Jacques C, Goulet A, Simoneau H, Ladouceur R (2018). Efficacy of a self-help treatment for at-risk and pathological gamblers. J Gambl Stud.

[15] Diskin KM, Hodgins DC (2009). Behaviour research and therapy a randomized controlled trial of a single session motivational intervention for concerned gamblers. Behav Res Ther.

[16] Hodgins DC, Currie SR, el-Guebaly N (2001). Motivational enhancement and self-help treatments for problem gambling. J Consult Clin Psychol.

[17] Hodgins DC, Currie SR, Currie G, Fick GH (2009). Randomized trial of brief motivational treatments for pathological gamblers: more is not necessarily better. J Consult Clin Psychol.

[18] Cowlishaw S, Merkouris S, Dowling N, Anderson C, Jackson A, Thomas S (2012). Psychological therapies for pathological and problem gambling. [Review]. Cochrane Database Syst Rev.

[19] Goslar M, Leibetseder M, Muench HM, Hofmann SG, Laireiter A-R (2017). Efficacy of face-to-face versus self-guided treatments for disordered gambling: a meta-analysis. J Behav Addict.

[20] Pallesen S, Mitsem M, Kvale G, Johnsen B, Molde H (2005). Outcome of psychological treatments of pathological gambling: a review and meta-analysis. Addiction.

[21] Carlbring P, Andersson G, Cuijpers P, Riper H, Hedman-Lagerlöf E (2018). Internet-based vs. face-to-face cognitive behavior therapy for psychiatric and somatic disorders: an updated systematic review and meta-analysis. Cogn Behav Ther.

[22] Carlbring P, Smit F (2008). Randomized trial of internet-delivered self-help with telephone support for pathological gamblers. J Consult Clin Psychol.

[23] Castrén S, Pankakoski M, Tamminen M, Lipsanen J, Ladouceur R, Lahti T (2013). Internet-based CBT intervention for gamblers in Finland: experiences from the field. Scand J Psychol.

[24] Myrseth H, Brunborg GS, Eidem M, Pallesen S (2013). Description and pre-post evaluation of a telephone and Internet based treatment programme for pathological gambling in Norway: a pilot study. Int Gambl Stud.

[25] Erevik EK, Pallesen S, Mohn M, Aspeland T, Vedaa Ø, Torsheim T (2020). The Norwegian remote intervention programme for problem gambling: short- and long-term outcomes.

[26] Dowling NA, Merkouris SS, Rodda SN, Smith D, Aarsman S, Lavis T (2021). GamblingLess: a randomised trial comparing guided and unguided Internet-based gambling interventions. J Clin Med.

[27] Hodgins DC, Cunningham JA, Murray R, Hagopian S (2019). Online self-directed interventions for gambling disorder: randomized controlled trial. J Gambl Stud.

[28] Cunningham JA, Hodgins DC, Mackenzie CS, Godinho A, Schell C, Kushnir V (2019). Randomized controlled trial of an Internet intervention for problem gambling provided with or without access to an Internet intervention for co-occurring mental health distress. Internet Interv.

[29] Wall H, Magnusson K, Berman AH, Bewick BM, Hellner C, Jayaram-lindström N, et al. Evaluation of a brief online self - help program for concerned. J Gambl Stud. 2021. 10.1007/s10899-021-10005-6 Springer US. 10.1007/s10899-021-10005-6 PMC8572834 33559778

[30] Stödlinjen (2019). Stödlinjens Årsrapport 2019.

[31] Ferris J, Wynne H (2001). The Canadian Problem Gambling Index: user manual.

[32] Wall H, Berman AH, Jayaram-Lindström N, Hellner C, Rosendahl I. Gambler clusters and problem gambling severity: a cluster analysis of Swedish gamblers accessing an online problem gambling screener. Psychol Addict Behav. 2021;35:102–12. Educational Publishing Foundation. 10.1037/adb0000674 32614206

[33] Rockloff MJ (2012). Validation of the consumption screen for problem gambling (CSPG). J Gambl Stud.

[34] Kroenke K, Spitzer RL, Williams JB (2001). The Phq-9. J Gen Intern Med.

[35] Suresh K (2011). An overview of randomization techniques: an unbiased assessment of outcome in clinical research. J Hum Reprod Sci.

[36] Snow G (2020). blockrand: randomization for block random clinical trials. R package version 1.5.

[37] Magnusson K, Nilsson A, Carlbring P. Modeling longitudinal gambling data: challenges and opportunities. PsyArXiv; 2019. Available from: psyarxiv.com/uvxk2.

[38] Smith VA, Neelon B, Preisser JS, Maciejewski ML (2017). A marginalized two-part model for longitudinal semicontinuous data. Stat Methods Med Res.

[39] Bürkner P (2017). Brms: an R package for bayesian multilevel models using Stan. J Stat Softw.

[40] Carpenter B, Gelman A, Hoffman MD, Lee D, Goodrich B, Betancourt M, et al. Stan: a probabilistic programming language. J Stat Softw. 2017;76 Available from: https://developer.apple.com/xcode/. Cited 2021 May 18. 10.18637/jss.v076.i01 PMC9788645 36568334

[41] Hodgins DC, Makarchuk K (2003). Trusting problem gamblers: reliability and validity of self-reported gambling behavior. Psychol Addict Behav.

[42] Holtgraves T (2009). Evaluating the problem gambling severity index. J Gambl Stud.

[43] Wickwire EM, Burke RS, Brown SA, Parker JD, May RK (2008). Psychometric evaluation of the National Opinion Research Center DSM-IV Screen for Gambling Problems (NODS). Am J Addict.

[44] Hodgins DC (2004). Using the NORC DSM screen for gambling problems as an outcome measure for pathological gambling: psychometric evaluation. Addict Behav.

[45] Hodgins D, Peden N, Makarchuk K (2004). Self-efficacy in pathological gambling treatment outcome: development of a gambling abstinence self-efficacy scale (GASS). Int Gambl Stud.

[46] Raylu N, Oei TPS (2004). The gambling urge scale: development, confirmatory factor validation, and psychometric properties. Psychol Addict Behav.

[47] Lundin A, Hallgren M, Balliu N, Forsell Y (2015). The use of alcohol use disorders identification test (AUDIT) in detecting alcohol use disorder and risk drinking in the general population: validation of AUDIT using schedules for clinical assessment in neuropsychiatry. Alcohol Clin Exp Res.

[48] Berman AH, Bergman H, Palmstierna T, Schlyter F (2005). Evaluation of the drug use disorders identification test (DUDIT) in criminal justice and detoxification settings and in a Swedish population sample. Eur Addict Res.

[49] Spitzer RL, Kroenke K, Williams JBW, Löwe B. A brief measure for assessing generalized anxiety disorder. Arch Intern Med. 2006;166:1092. Available from: http://archinte.jamanetwork.com/article.aspx?doi=10.1001/archinte.166.10.1092. 10.1001/archinte.166.10.1092 16717171

[50] Skevington SM, Lotfy M, O’Connell KA (2004). The World Health Organization’s WHOQOL-BREF quality of life assessment: psychometric properties and results of the international field trial a report from the WHOQOL Group. Qual Life Res.

[51] Nilsson A, Magnusson K, Carlbring P, Andersson G, Hellner C (2020). Behavioral couples therapy versus cognitive behavioral therapy for problem gambling: a randomized controlled trial. Addiction.

[52] Carr MM, Ellis JD, Ledgerwood DM (2018). Suicidality among gambling helpline callers: a consideration of the role of financial stress and conflict. Am J Addict.

[53] Weinstock J, Scott TL, Burton S, Rash CJ, Moran S, Biller W (2014). Current suicidal ideation in gamblers calling a helpline. Addict Res Theory.

[54] Jonas B, Leuschner F, Eiling A, Schoelen C, Soellner R, Tossmann P (2020). Web-based intervention and email-counseling for problem gamblers: results of a randomized controlled trial. J Gambl Stud.

[55] Blaszczynski A, Ladouceur R, Goulet A, Savard C (2006). ‘How much do you spend gambling?’: ambiguities in questionnaire items assessing expenditure. Int Gambl Stud.

[56] Carlbring P, Jonsson J, Josephson H, Forsberg L (2010). Motivational interviewing versus cognitive behavioral group therapy in the treatment of problem and pathological gambling: a randomized controlled trial. Cogn Behav Ther.

[57] Casey LM, Oei TPS, Raylu N, Horrigan K, Day J, Ireland M (2017). Internet-based delivery of cognitive behaviour therapy compared to monitoring, feedback and support for problem gambling: a randomised controlled trial. J Gambl Stud.

[58] Miller WR, Rollnick S (2002). Motivational interviewing: preparing people for change.

